# Extracellular Vesicles from Fresh and Dried Plants—Simultaneous Purification and Visualization Using Gel Electrophoresis

**DOI:** 10.3390/ijms20020357

**Published:** 2019-01-16

**Authors:** Eric Woith, Matthias F. Melzig

**Affiliations:** Institute of Pharmacy—Pharmaceutical Biology, Dahlem Center of Plant Sciences, Freie Universitaet Berlin, Koenigin-Luise-Str. 2+4, D-14195 Berlin, Germany; e.woith@fu-berlin.de

**Keywords:** extracellular vesicles, apoplastic fluid, dried plants, purification, electrophoresis

## Abstract

Although animal-derived extracellular vesicles (EVs) are moving increasingly into scientific focus, EVs from other kingdoms remain underestimated and our knowledge of them is still expandable, probably due to the lack of an easy and broadly executable isolation, purification and visualization method. Using differential centrifugation with subsequent agarose gel electrophoresis, we were able to simplify the terms of EV isolation. EVs from *Nicotiana tabacum* L., *Vinca minor* L., and *Viscum album* L. were purified, even though they did not migrate into the gel matrix. If 3,3- Dihexyloxacarbocyanine iodide (DiOC${}_{6}$) is added to the specimen in excess, membranous components can already be detected by eye, or with higher sensitivity, using a UV transilluminator. The sample preparation can be adjusted to the EV species of interest. Moreover, EVs are separated from small charged contaminants and dye excess, because these impurities can pass the gel matrix, while EVs themselves are retained in the pocket. Significantly, we isolated EVs from dried plant material, which is—to our knowledge—the first proof that EVs are stable enough to overcome the drying process of plant material.

## 1. Introduction

Extracellular vesicles (EVs) are attracting increasing attention. They have been observed in all empires of life—archaea, bacteria, and eukaryotes [1]. This ubiquity indicates the high evolutionary importance of EVs. EV subpopulations are usually classified by their size or origin [2]. The sub-population of exosomes is the smallest and most investigated class among EVs. In particular, mammalian cell-derived exosomes are well characterized. In several studies, plant-derived EVs (PEVs) have been named “exosome-like” due to their similar morphology and density compared to mammalian exosomes [3,4,5,6]. Since exosomes are defined to originate from multivesicular bodies (MVBs), it has been demonstrated that plant cells release EVs being genuine exosomes rather than just “exosome-like” [2,7]. But nomenclature of PEVs is still evolving. An alternative method for PEV designation is the distribution into a microvesicle and a nanovescile fraction, since bulk PEV preparations show broad size distributions ranging from 20–500 nm [8]. PEV sizes appear to be species-specific, with medians between 100–400 nm [8,9]. Due to the inhomogeneous nomenclature, we desist from further specification of the vesicles we isolated.

Human exosomes are thought to be tumor markers and thereby probably useful in cancer diagnostics. Furthermore, promising indications for therapeutic uses of EVs have been found. For instance, positive effects of PEVs were shown in cancer or colitis treatment [3,5,10]. EVs are assumed to be potent agents in cross-species and even in cross-kingdom regulation processes [11,12,13]. Thus, they are of particular interest as vehicles for drug delivery [10,14,15]. For this purpose, large amounts of vesicles are required. PEVs are biocompatible and biodegradable and therefore plants are interesting factories producing raw material for innovative therapeutic agents [16]. However, PEVs are still barely characterized, while a broad set of information—including knowledge of the vesicular shell and cargo—is available on mammalian EVs. Several human exosome marker proteins are commonly used for purification and identification, especially the transmembrane tetraspanins CD9, CD63, and CD81 [17,18,19]. Specific markers for PEVs, consistent in all species in the plant kingdom, are unknown so far. Fortunately, first steps towards this direction have been made and some interesting proteins identified, such as Patellins 1–3 [2,8], Penetration 1 [2], Clathrin heavy chain [2,3,5,8], and heat shock proteins [2,3,5,8,16].

Our time- and cost-efficient method for EV purification and detection using agarose gel electrophoresis can possibly form the basis for further characterization, to gain more information on PEVs.

## 2. Results and Discussion

Using differential centrifugation, PEVs were concentrated and soluble protein contaminants reduced to a minimum in the final 50,000× *g* centrifugation step. Dynamic light scattering (DLS) analysis of resuspended 50,000× *g* pellets showed broad size distributions for all PEV isolations. Mean diameters of PEV preparations from dried herbs were determined as follows using DLS: *Vinca minor* L. 380 ± 200 nm and *Viscum album* L. 280 ± 115 nm. For *Nicotiana tabacum* L. two cohorts of particles were found with 70 ± 20 nm and 520 ± 170 nm.

**Figure 1:**
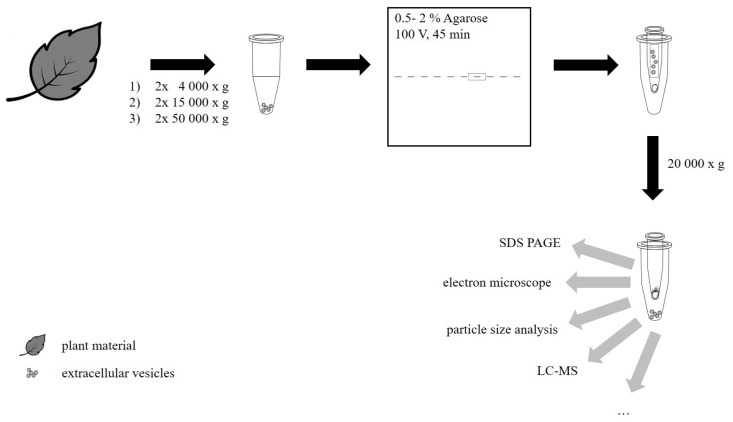
Workflow of PEV isolation, agarose gel electrophoresis, recovery, and possible further analysis (Note: Currently, we did perform SDS PAGE after agarose gel electrophoresis. Further methods mentioned are suggestions, which we are planning to perform in future investigations.)

For further purification, 50,000× *g* pellets were applied on agarose gels, soluble contaminants separated from the vesicles, and the gel matrix removed by centrifugation. Figure 1 shows the workflow of our method. The displayed analytical methods are suggestions, since we verified EV recovery just by sodium dodecyl sulfate polyacrylamide gel electrophoresis (SDS PAGE).

**Figure 2:**
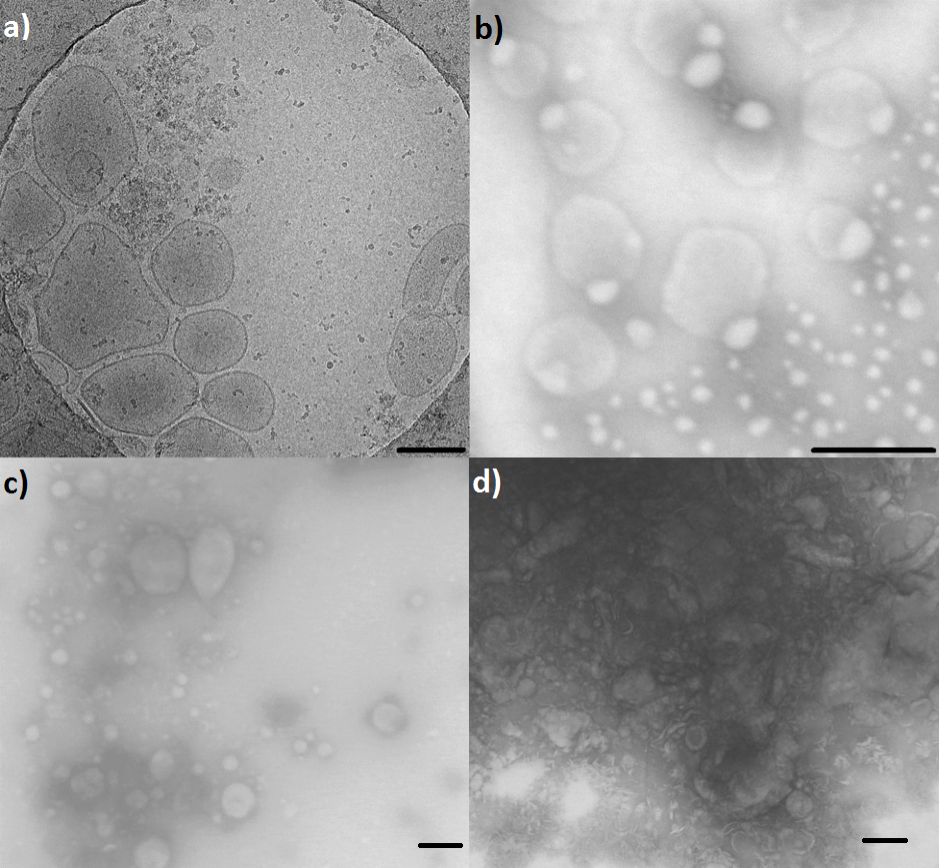
Electron microscopy of EV isolates. (**a**) Cryo-TEM image of *N. tabacum* PEVs from apoplastic fluid (APF). (**b**) TEM image of *N. tabacum* PEVs from dried herb. (**c**) TEM image of *V. album* PEVs from dried herb. (**d**) TEM image of *V. minor* PEVs from dried herb. Scale bar = 200 nm.

Figure 2 shows electron microscopy (EM) images of the investigated PEVs. Discrepancies of the vesicle sizes between DLS and EM data result from shrinking effects, due to the drying process during preparation for transmission electron microscopy (TEM). However, Cryo-TEM imaging of *N. tabacum* supported DLS data.

**Figure 3:**
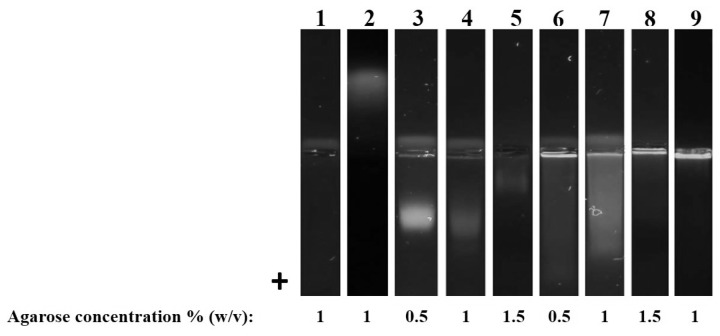
Agarose gel electrophoresis of EVs: (**1**) DiOC${}_{6}$ working concentration (**2**) DiOC${}_{6}$ 10× working concentration (**3**–**5**) exosome standard (**6**–**8**) 50,000× *g* pellet *V. album* (**9**) 50,000× *g* pellet *V. minor*.

As shown in Figure 3, unbound 3,3${}^{\prime }$-dihexyloxacarbocyanine iodide (DiOC${}_{6}$) was not detectable in working concentrations. Nevertheless, when the concentration was elevated 10 folds, the membrane dye migrated towards the cathode. DiO dyes are known to be weakly fluorescent in aqueous solutions, while fluorescence intensity increases after membrane incorporation [20]. That is why dye excess is not detectable in working concentrations. However, as observed when using higher DiOC${}_{6}$ concentrations, unbound dye is removed from the pocket in cathode direction. All investigated EVs moved in direction of the anode (PEVs caused a deformation of the pocket towards the anode).

Since human exosomes are smaller than PEVs (30–150 nm), they were able to migrate into the gel, forming diffuse bands. Meanwhile, plant derived EVs were mainly retained in the pockets, whereas comet-like tails have been observed. This tailing is probably a result of the broad PEV size distribution. While a smaller proportion of vesicles fits into the gel pores, larger vesicles are excluded. The cut off size of the gels is determined by agarose concentration and can be easily adapted to the desired particle size that shall be investigated. Small charged suspended or soluble contaminants are anyway separated from PEVs, as well as DiOC${}_{6}$ excess. Large impurities, such as apoptotic bodies or larger microvesicles, were removed during differential centrifugation.

EVs were recovered from agarose gels by excising with a surgical blade and removing them from the gel by centrifugation, according to the DNA extraction of Sun et al., 2012 [21]. Investigating whether protein contamination is really separated from EVs during agarose gel electrophoresis, we added 10 μg bovine serum albumin (BSA, Cat. No. 0163.2, Carl Roth, Karlsruhe, Germany) prior to the application on agarose gel, imitating a protein contaminant. When we added BSA and DiOC${}_{6}$ together as blank, fluorescence was detectable in anode direction. Since DiOC${}_{6}$ alone would head towards the cathode, BSA must have bound the dye resulting in a negatively charged adduct. Applying 50,000× *g* pellets or supernatants to the gel resulted in blurry fluorescence mainly in anode direction, which were obviously soluble proteins interacting with the fluorescence dye. Due to the relatively large size of PEVs they remained in the pocket, while contaminants were electrophoretically separated.

**Figure 4:**
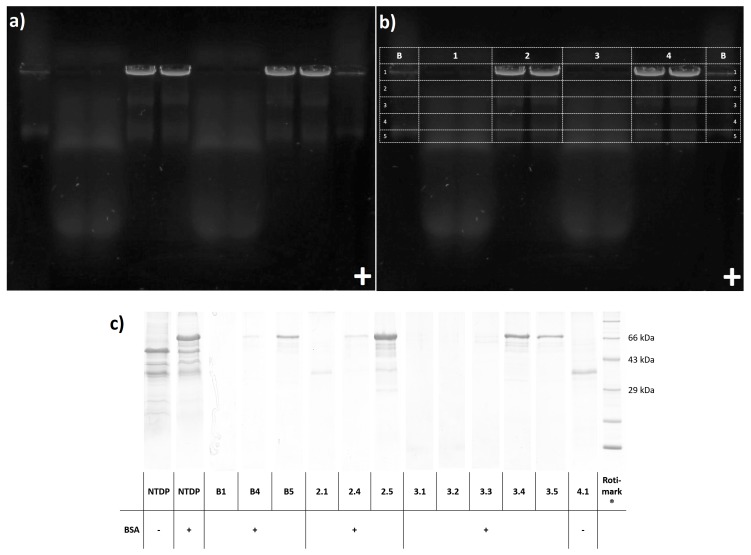
(**a**) agarose gel of dried *N. tabacum* 50,000× *g* pellet (NTDP) and supernatant (NTDS) with and without BSA added, and BSA-blank (for application order see b) at 254 nm with 530 nm band filter (**b**) pattern of agarose gel slicing: B- BSA-blank(DiOC${}_{6}$+BSA) 1- NTDS 2- NTDP+BSA 3- NTDS+BSA 4- NTDP (**c**) SDS PAGE of NTDP, NTDP+BSA, and NTDS before and after agarose gel electrophoresis (selected agarose gel slices after TCA precipitation).

This finding was supported by slicing the agarose gel into sections with subsequent trichloroacetic acid (TCA) precipitation and SDS PAGE (see Figure 4). While BSA (66 kDa) was not detectable in the pocket cut outs (line B section 1, line 2 section 1, and line 3 section 1), we did recover albumin from the fluorescing zone of BSA-blank (line B sections 4 and 5) and from the corresponding migration distances in 50,000× *g* pellet (line 2 sections 4 and 5) and supernatant (line 3 sections 4 and 5).

SDS PAGE of pellets (pocket cut outs line 2 section 1 and line 4 section 1) showed a characteristic protein band at ~35 kDa. The added BSA (line 2 section 1) was not recovered, proving that PEVs can be recovered from the gel and are indeed purified from soluble charged protein contamination.

## 3. Materials and Methods

### 3.1. Plant Material

*Nicotiana tabacum* L. was provided by the Botanical Garden Berlin (accession number 107-01-95-14) and either investigated freshly or air dried (at room temperature for several weeks). *Vinca minor* L. and *Viscum album* L. were purchased as dried herbs from Alfred Galke GmbH, Bad Grund, Germany. The received plant material was analytically certified and used as provided. *V. minor*: Cat. No. 134402, Lot: 27105, origin: Romania, authorized 3 May 2018. *V. album*: Cat. No. 136202, Lot: 30876, origin: Serbia, authorized 27 November 2017.

### 3.2. PEV Isolation

PEVs were isolated from apoplastic fluid (APF) and from dried plant material of *N. tabacum*, *V. minor*, and *V. album*. For the isolation from APF, we modified the method of Rutter and Innes 2016 [2] to our lab conditions. In brief, plant leaves were collected and washed, then infiltrated under vacuum with vesicle isolation buffer (VIB: 20 mM MES, 2 mM CaCl${}_{2}$, 100 mM NaCl, pH 6.0). APF was collected by carefully rolling the infiltrated leaves, to fit them into syringes, which were then placed into 50 mL reaction tubes and centrifuged at 4000× *g* twice for 20 min.

For the isolation of PEVs from dried plant material, we incubated the herbs for 24 h in VIB at room temperature under gentle shaking, to reconstitute EVs. Rough material was removed by decanting. To remove particulate impurities, as well as large EV populations, the supernatant, respectively APF, was then differentially centrifuged.

Apoptotic bodies (1000–5000 nm) can be pelleted together with large particles and cells at low centrifugal forces. Intermediate sedimentation speed was subsequently applied for separation of microvesicles (100–1000 nm) [2,15]. Thus, for separation of larger vesicle species and debris, specimens were successively centrifuged twice at 4000× *g* and twice at 15,000× *g*, 20 min each, the pellets were discarded. DiOC${}_{6}$ (Sigma Aldrich, St. Louis, USA) was added to the 15,000× *g* supernatant in excess, staining the vesicle membrane. For sedimentation of nanosized PEVs, samples were spun at 50,000× *g* for 90 min. After 50,000× *g* centrifugation, the supernatant was separated, the pellet resuspended in VIB and the 50,000× *g* centrifugation step repeated, washing the EVs from soluble contaminants. All centrifugations were performed at 4 ${}^{\circ }$C, using Beckman Allegra X 30 R and Avanti J-26 S XP centrifuges (Beckman Coulter, Brea, CA, USA).

### 3.3. Agarose Gel Electrophoresis and EV Recovery

The final 50,000× *g* pellet was resuspended in VIB and loading dye added (50% (*v*/*v*) glycerol, 0.05% (*w*/*v*) bromophenol blue in TBE-buffer). 25 μL of the mix were applied to the pockets of 0.5, 1.0, and 1.5% (in TBE-buffer) agarose gels. Agarose was purchased from SERVA Electrophoresis GmbH, Heidelberg, Germany. As positive control, we used human colon carcinoma cell line exosome standard (Cat-Code: HBM-COLO-30, HansaBioMed Life Sciences Ltd., Tallinn, Estonia). For blank, we exchanged exosome standard by VIB. 1 μL DiOC${}_{6}$ (1 mM in Methanol) was added together with the loading dye, in case of positive control and blank. Electrophoresis was conducted in TBE-buffer at 100 V for 60 min. EVs were visualized in the gel, using a UV transilluminator (Biostep GmbH, Burkhardtsdorf, Germany) at 254/366 nm and a 530 nm band filter on the camera objective (Canon EOS 700D, Canon Inc., Tokyo, Japan).

Recovery of EVs from agarose gels was performed according to the DNA extraction method of Sun et al., 2012 [21]. Gel sections, which should be further investigated, were excised using a surgical blade. Gel slices were then placed into 0.5 mL reaction tubes, which were punctured at the bottom. To retain agarose in the upper tube, a small cotton ball was placed inside. Finally, the 0.5 mL tubes were inserted into 1.5 mL tubes and centrifuged at 20,000× *g* for 20 min at 4 ${}^{\circ }$C.

### 3.4. SDS PAGE

For further analysis by SDS PAGE, proteins were precipitated by adding 1 volume of 8.7 M TCA solution to 4 sample volumes. Samples were then incubated for 30 min at 4 ${}^{\circ }$C and centrifuged at 20,000× *g* for 10 min at 4 ${}^{\circ }$C. The supernatant was discarded and the pellet washed in cold (−20 ${}^{\circ }$C) acetone. Samples were centrifuged again and the acetone washing of the pellet repeated. The final acetone supernatant was removed and the pellets air dried for approximately 30 min. Dry pellets were resuspended in 20 μL VIB. Discontinuous SDS PAGE was conducted after the instructions by Jansohn and Rothaemel 2012 [22] using 15% (*w*/*v*) polyacrylamide resolving gel and 5% (*w*/*v*) polyacrylamide stacking gel on top. 25 μL of each sample were added to gel pockets after mixing with 4× Laemmli buffer (Bio-Rad Laboratories, Inc., Hercules, CA, USA) with β-mercaptoethanol added and denaturing for 15 min at 95 ${}^{\circ }$C. Electrophoresis was performed at 175 V for approximately 60 min, until bromophenol reached the bottom of the gel. After electrophoresis, gels were immediately transferred into the staining solution. According to Hoffman et al., 1988 [23], gels were stained overnight in 0.1% (*w*/*v*) Coomassie Brilliant Blue G-250, 2% (*v*/*v*) phosphoric acid, 10% (*w*/*v*) ammonium sulfate, and 20% (*v*/*v*) methanol. The next day, gel matrices were destained using 25% (*v*/*v*) methanol.

### 3.5. Dynamic Light Scattering

DLS analyses were performed using a Nicomp ZLS Z3000 (Entegris Inc., Billerica, MA, USA) and semi-micro acryl cuvettes (Sarstedt, Nümbrecht, Germany). Samples were equilibrated at 23 ${}^{\circ }$C for 10 min followed by 5 measurements, 1 min each.

### 3.6. Electron Microscopy

The occurrence of EVs before and after agarose gel electrophoresis was confirmed by EM. For TEM analysis we used the scanning electron microscope Hitachi SU 8030 in TEM mode (Hitachi Ltd., Tokyo, Japan). Samples were prepared using the protocol of Rutter and Innes 2016 [2], without glow discharging grids and replacing uranyl acetate by Uranlyess (Science Services GmbH, Munich, Germany). 5 μL of each sample were placed on 300 mesh formvar and carbon coated copper grids and incubated 5 min. Grids were then rinsed and negatively stained, pipetting 100 μL Uranyless (Science Services GmbH, Munich, Germany) across the grid surface. Fluid excesses were carefully blotted using Kimwipe and the grids dried overnight in a desiccator. Imaging was performed using 30 kV acceleration voltage.

For Cryo-TEM imaging, freshly prepared EVs from *N. tabacum* APF were plunge frozen vitrifying VIB, by use of a Vitrobot (ThermoFisher Scientific Inc., Waltham, MA, USA). The major advantage of this method is that the formation of artifacts, e.g., caused by additives or drying, is reduced to a minimum and the native structure of the sample is preserved [24,25]. Samples were observed on Talos™ Arctica (ThermoFisher Scientific Inc., Waltham, MA, USA), without the addition of any fixatives or contrasting agents. Imaging was performed at 200 kV and a primary magnification of 28 k using the microscopes’ low-dose protocol. The defocus was chosen to be 4.9 μ to create sufficient phase contrast. Images were recorded by a 4 k Falcon IIIC direct electron detector (ThermoFisher Scientific Inc., Waltham, MA, USA) at full resolution.

## 4. Conclusions and Future Perspectives

PEVs appear to be very stable packaging for their cargo, since they even overcome drying, which means severe osmotic stress. The uprising interest on EVs created the necessity of a simple and handy method to isolate, purify and visualize them. We established an easy alternative method for EV visualization with simultaneous purification. Our protocol might be easier than density gradient centrifugation or size exclusion chromatography. In addition, our method is feasible with basic biochemical equipment. Furthermore, distinct EV subpopulations can be investigated by simply adapting differential centrifugation and agarose concentration.

Further investigations are intended to identify PEV cargos and protein patterns or even markers. Therefore, we are planning a proteomic screening of PEVs from diverse species, including the direct comparison of vesicles prepared from fresh, as well as from dried material of the same plant.

## Abbreviations

The following abbreviations are used in this manuscript:

APF	apoplastic fluid
BSA	bovine serum ablumine
DiOC${}_{6}$	3,3${}^{\prime }$-dihexyloxacarbocyanine iodide
DLS	dynamic light scattering
EM	electron microscopy
EVs	extracellular vesicles
MVB	multivesicular body
PEVs	plant-derived extracellular vesicles
SDS PAGE	sodium dodecyl sulfate polyacrylamide gel electrophoresis
TCA	trichloroacetic acid
TEM	transmission electron microscopy
VIB	vesicle isolation buffer
